# Comparative impact of antiretroviral drugs on markers of inflammation and immune activation during the first two years of effective therapy for HIV-1 infection: an observational study

**DOI:** 10.1186/1471-2334-14-122

**Published:** 2014-03-04

**Authors:** Suhaib Hattab, Amelie Guihot, Marguerite Guiguet, Slim Fourati, Guislaine Carcelain, Fabienne Caby, Anne-Geneviève Marcelin, Brigitte Autran, Dominique Costagliola, Christine Katlama

**Affiliations:** 1 INSERM, UMR_S 1136, Pierre Louis Institute of Epidemiology and Public Health, Paris F-75013, France; 2 Sorbonne Universities, UPMC Univ Paris 06, UMR_S 1136, Pierre Louis Institute of Epidemiology and Public Health, Paris F-75013, France; 3 INSERM, UMR_S 1135, CIMI, Paris F-75013, France; 4 Sorbonne Universities, UPMC Univ Paris 06, UMR_S 1135, CIMI, Paris F-75013, France; 5 AP-HP, Hôpital Pitié-Salpêtrière, Département d’immunologie, Paris F-75013, France; 6 AP-HP, Hôpital Pitié-Salpêtrière, Service de virologie, Paris F-75013, France; 7 AP-HP, Hôpital Pitié-Salpêtrière, Service des maladies infectieuses et tropicales, Paris F-75013, France

**Keywords:** HIV, cART, Immune activation, Inflammation, Markers

## Abstract

**Background:**

Few studies have compared the impact of different antiretroviral regimens on residual immune activation and inflammation with discordant results. Aim of the study was to investigate the impact of various antiretroviral regimens on markers of immune activation and inflammation during the first two years of effective therapy.

**Methods:**

We studied HIV-infected antiretroviral-naïve patients who began cART with either abacavir/lamivudine or tenofovir/emtricitabine, combined with ritonavir-boosted lopinavir (LPV/r), atazanavir (ATV/r) or efavirenz (EFV). All the patients had a virological response within 6 months, which was maintained for 2 years with no change in their ART regimen. C-reactive protein (hs-CRP), interleukin-6 (IL-6), soluble CD14 (sCD14), monokine induced by interferon-γ (MIG) and interferon-γ-inducible protein-10 (IP-10) were measured in stored plasma obtained at cART initiation and 24 months later. Mean changes from baseline were analyzed on log_e_-transformed values and multivariable linear regression models were used to study the effect of the treatment components, after adjusting for factors that might have influenced the choice of ART regimen or biomarker levels. Differences were expressed as the mean fold change percentage difference (Δ).

**Results:**

Seventy-eight patients (91% males) with a median age of 43 years met the inclusion criteria. Their median baseline CD4 cell count was 315/mm^3^ and HIV-1 RNA level 4.6 log_10_ copies/ml. During the 2-years study period, IL-6, IP-10 and MIG levels fell significantly, while hs-CRP and sCD14 levels remained stable. IP-10 and MIG levels declined significantly less strongly with ATV/r than with EFV (IP-10Δ -57%, p = 0.011; MIGΔ -136%, p = 0.007), while no difference was noted between LPV/r and EFV. The decline in IL-6 did not differ significantly across the different treatment components.

**Conclusions:**

After the first 2 years of successful cART, IL-6, IP-10 and MIG fell markedly while hs-CRP and sCD14 levels remained stable. The only impact of ART regimen was a smaller fall in markers of immune activation with ATV/r than with EFV. Our results suggest that these markers could be worthwhile when evaluating new antiretroviral drugs.

## Background

Antiretroviral therapy (ART) leads to a dramatic reduction in HIV-related morbidity and mortality [1] and currently suppresses viral replication in the vast majority of compliant patients [2]. Most treatment guidelines recommend early initiation of ART, based mainly on nucleoside reverse transcriptase inhibitors (NRTI) combined with non-nucleoside reverse transcriptase inhibitors (NNRTI) or protease inhibitors (PI) [3,4]. However, despite viral suppression and quantitative immune restoration in most patients, signs of immune activation and inflammation persist [5,6]. This may be due in part to ongoing low-level viral replication [7], the coinfection with other chronic viruses such as cytomegalovirus and Epstein-Barr virus [8,9] and to the consequences of mucosal immune dysfunction that is characterized by a profound depletion of CD4 + T-cells during the early acute infection, and a progressive loss of the ability to maintain the intestinal barrier function, allowing translocation of the intestinal microbial flora into the systemic circulation which induces immune activation and inflammation cascades [10]. An additional factor that may contribute to this persistence is the perturbation of immune regulatory mechanisms, such as regulatory T-cells or the immune regulatory cytokines such as interleukin-10 [11].

By comparison with HIV-uninfected individuals, several studies have shown an excess of comorbidities in HIV-infected patients on virologically effective treatment, including metabolic disorders [12], decreased bone mineral density with an increased risk of fractures [13], an elevated cardiovascular risk [14], and cancers [15]. These comorbidities have been attributed to a higher prevalence of standard risk factors such as smoking in HIV-infected patients, as well as to past or present HIV-induced immune depression reflected by the CD4 cell nadir or the CD4/CD8 cell ratio [16,17], and to the effect of some antiretroviral drugs [12,18,19]. In addition, elevated levels of markers of inflammation, such as interleukin-6 (IL-6) and C-reactive protein (CRP), have been linked to increased morbidity and mortality in both the general and HIV-infected populations [20-22]. The SMART study was the first to demonstrate that mortality in this setting is linked to HIV replication, inflammatory markers such as IL-6 and soluble CD14 (sCD14), and coagulation markers such as D-dimers [23]. Since then, several studies have shown an association between plasma markers of inflammation and the risk of non AIDS-defining events [24,25]. In the ALLRT cohort, elevated levels of IL-6, sCD14, D-dimers and soluble tumor necrosis factor receptors (sTNFR1 and sTNFR2), both prior to and during ART, were associated with the occurrence of non AIDS-related morbidities and death [26].

Now that ART is recommended for all HIV-infected patients, and life expectancy is greatly prolonged by suppressive ART despite the lack of viral eradication, it is important to compare the potential impact of different antiretroviral strategies on residual immune activation and inflammation. There are some arguments to suggest that different drugs from different classes may have different impact on immune activation and inflammation. In the Spiral study, a switch from PI-based therapy to a raltegravir-containing regimen in patients with suppressed viremia led to a decrease in biomarkers associated with inflammation, insulin resistance and hypercoagulability [27].

Few studies have examined the impact of first-line ART on biomarkers of inflammation and immune activation, and they have given discordant results [28-31]. These discordant results might reflect differences in baseline HIV disease status, and/or differences in the virological response, and/or treatment switches.

We therefore explored the impact of different first-line ARV regimens on soluble markers of inflammation and immune activation during the first two years of effective therapy, while controlling for potential confounders such as HIV replication, and changes in the initial regimen.

## Methods

### Study design

We compared the impact of commonly used first-line antiretroviral drugs on soluble markers of inflammation and immune activation, while controlling for potential confounders. In order to avoid the influence of previous ARV exposure, active viral replication and treatment switches, we restricted our analysis to a homogenous group of treatment-naive HIV-infected patients who had experienced a rapid and persistent virological response and remained on their initial regimen for 2 years. Because the biomarkers of interest were not part of the patients' routine biological monitoring, we restricted our analysis to patients for whom plasma samples stored at ART initiation and 2 years later were available. The analyses were also adjusted for baseline characteristics that might have influenced the choice of cART regimen or affected biomarker levels, such as the age, smoking status, CD4 cell count, prior AIDS-defining events, plasma HIV-1 viral load, and hepatitis virus coinfection.

### Study population

All HIV-infected patients receiving care in the Infectious Diseases department of Pitié-Salpêtrière Hospital (Paris, France) have their clinical, biological and therapeutic findings recorded prospectively in standardized electronic medical records (NADIS). Biological data obtained in the hospital, such as HIV RNA levels and immunological parameters, are directly imported from the laboratory computer system, thus minimizing collection bias. The quality of the database is ensured by automated checks during data capture, and by regular controls and annual assessments. Routine blood tests are performed at each hospital visit, and residual plasma is stored frozen, being identified by a serial number.

All HIV-1-infected patients who started first-line cART between January 2006 and December 2009 were screened for eligibility, using the hospital database. Patients were included in this study if they received either tenofovir-emtricitabine (TDF/FTC) or abacavir-lamivudine (ABC/3TC), combined with efavirenz or with a ritonavir-boosted protease inhibitor (atazanavir (ATV/r) or lopinavir (LPV/r)). In order to control for potential causes of inflammation/activation due to persistent plasma viral replication, we only studied patients who had a rapid and persistent virological response, defined by a plasma HIV-1 viral load (VL) below 400 copies/mL at 6 months and below 50 copies/mL at 24 months, with no values above 1000 copies/ml between month 6 and month 24. Other eligibility criteria included no change in antiretroviral therapy throughout the 24 months of the study, and the availability of frozen plasma samples obtained at baseline (D0) and month 24 (M24). The study was approved by the Pitié-Salpêtrière institutional review board, and the patients were asked to give their written consent to the use of their medical information and plasma samples, as required by French law.

### Sample collection and plasma soluble markers measurements

We selected markers of inflammation and immune activation that can be reliably measured in frozen plasma. We evaluated IL-6 and hs-CRP as markers of inflammation, soluble CD14 (sCD14) as a marker of monocyte activation, and interferon-γ-inducible protein 10 (IP-10) and monokine induced by interferon-γ (MIG) as markers of T-lymphocyte and macrophage activation.

Enzyme-linked immunosorbent assays (ELISA) were used according to manufacturer’s instructions to quantify IL-6, sCD14 (R&D Quantikine®, HS600B and DC140 respectively) and hs-CRP (Calbiotech®, CR120C). IP-10 and MIG levels were determined on thawed diluted plasma with Cytometric Bead Array kits (BD™ CBA) on a BD FACS Canto I according to manufacturer’s instructions. Coefficients of variation were 6.9-7.8% for IL-6, 7.9–8.5% for hs-CRP, 4.8–7.4% for sCD14, 4% for IP-10 and 9–13% for MIG. Plasma samples were allowed to thaw for 50 minutes at room temperature before centrifugation for 5 minutes at 1000 rpm, followed by distribution into Eppendorf tubes in the amounts required for each assay kit. Plasma was diluted as recommended by the kit manufacturers. Standards provided with the kits were measured in duplicate and the mean value was used as reference. Samples with values higher than the highest standard value were further diluted and retested. D0 and M24 samples from each patient were tested in the same run.

### Statistical analysis

Two NRTI backbone combinations (TDF/FTC vs ABC/3TC) and three third agents (LPV/r, ATV/r vs EFV) were compared in a factorial design. Baseline characteristics were compared between treatment groups by using the Wilcoxon and chi-squared tests in order to identify variables associated with the choice of treatment. Because the marker values were not normally distributed, they were log_e_-transformed for analysis. For each marker, changes between D0 and M24 (mean fold change) were expressed as the geometric mean of their ratio after log_e_ transformation. A paired one-sample *t* test was used to identify significant differences in the overall changes in each marker.

Linear regression models were used to investigate the impact of the different NRTI backbones and the different third agents on the biomarker changes. The results are expressed as the estimated percentage difference between the mean fold changes observed with a given drug, using TDF/FTC and EFV as the reference groups for the comparison. Relationships between baseline covariables and changes in each biomarker were examined in univariable linear regression models. These covariables were sex, age, body mass index, smoking status, hepatitis B or C virus (HBV or HCV) coinfection, prior AIDS-defining events, and the pre-ART CD4 cell count and viral load. Baseline covariables associated with changes in at least one biomarker (p < 0.10) and viral blips above 50 copies/ml between M6 and M24 were retained in all multivariable linear regression models in order to control for factors that might have influenced the choice of cART regimen or affected biomarker levels. Age and smoking status were included in the multivariable model since these variables are known to influence marker levels [32,33]. Interaction terms between the NRTI backbone and the third agent were tested for each marker. Sensitivity analyses were conducted, excluding patients with hepatitis virus coinfection. Statistical analyses were run on STATA 12 software, and p values <0.05 were considered statistically significant.

## Results

### Baseline characteristics

Between January 2006 and December 2009, a total of 539 patients began first antiretroviral therapy and remained under care over two years in our department. Of those, 370 patients had a rapid and persistent virological response over the two years. Of them, 280 patients who began therapy with abacavir/lamivudine or tenofovir/emtricitabine plus efavirenz, atazanavir/r, lopinavir/r or fosamprenavir/r were eligible for the study. The remaining 90 patients were not considered since they either received no longer recommended antiretroviral therapy such as combivir and invirase (n = 64) or were included in protocols evaluating new drugs such as darunavir and rilpivirine. Frozen plasma samples at baseline and after two years were available for 149 patients. Characteristics of patients who had stored plasma were not different from those did not have it for age, CD4 cell count, HIV-RNA level, AIDS defining events and for prescribed antiretroviral therapy. Two patients who withheld their consent were excluded. Among the remaining 147 patients, 78 remained on the same antiretroviral regimen throughout the 2-year study period and comprised the study population. Modifications in first ART regimen were due to side effects, drug toxicity, or switch to newly available drugs.

The NRTI backbone consisted of TDF/FTC in 61 patients (78%) and ABC/3TC in 17 patients. The third agent was EFV in 36 patients (46%), ATV/r in 27 patients (35%) and LPV/r in 15 patients. At baseline, the median viral load was 4.6 log_10_ copies/mL, the median CD4 cell count was 315/mm^3^ and the median CD4/CD8 cell ratio was 0.30. Seven patients (9%) had an AIDS-defining event. Four patients were HCV-PCR-positive and two were HBsAg-positive.

Baseline characteristics were well balanced across the treatment groups, with the following exceptions. As shown in Table 1, patients prescribed LPV/r had the lowest median CD4 cell count (177 cells/mm^3^), a value significantly different from the median count in patients prescribed ATV/r (330 cells/mm^3^) or EFV (342 cells/mm^3^) (p = 0.020). Patients prescribed TDF/FTC had a lower median CD4 cell count than patients prescribed ABC/3TC (304 versus 431 cells/mm^3^; p = 0.008). Four patients prescribed LPV/r had an AIDS-defining event, compared to only one patient prescribed ATV/r and two patients prescribed EFV (p = 0.030). All the patients with hepatitis virus coinfection were prescribed LPV/r (p < 0.001).

**Table 1:** Baseline characteristics of the patients

**N (%) or median (interquartile range)**						
	**Total n = 78**	**TDF/FTC n = 61**	**ABC/3TC n = 17**	**EFV n = 36**	**ATV/r n = 27**	**LPV/r n = 15**
**Sex: Male**	71 (91%)	56 (91%)	15 (88%)	34 (94%)	22 (81%)	15 (100%)
**Age, years**	43 (34-47)	44 (35-49)	38 (32-43)	42 (34-46)	44 (35-48)	42 (33-55)
**Body mass index**	23 (22-24)	23 (22-25)	22 (21-24)	23 (22-25)	22 (21-25)	22 (21-24)
**Current smokers**	28 (36%)	25 (41%)	3 (18%)	14 (39%)	6 (22%)	8 (53%)
**Viral hepatitis coinfection**	6 (8%)	5 (8%)	1 (6%)	0	0	6 (40%)
**Prior AIDS**	7 (9%)	5 (8%)	2 (12%)	2 (6%)	1 (4%)	4 (27%)
**CD4/mm**^**3**^	315 (217-409)	304 (190-384)	431 (330-512)	342 (262-402)	330 (263-446)	177 (72-299)
**CD8/mm**^**3**^	959 (660-1345)	949 (656-1335)	1053 (762-1468)	981 (774-1240)	1111 (646-1506)	709 (454-1109)
**CD4/CD8 ratio**	0.30 (0.19-0.46)	0.28 (0.17-0.41)	0.49 (0.22-0.59)	0.33 (0.19-0.46)	0.29 (0.24-0.49)	0.16 (0.13-0.38)
**Viral load (log**_**10 **_**copies/mL)**	4.6 (4.1-5.2)	4.7 (4.3-5.2)	4.4 (3.6-4.9)	4.8 (4.3-5.2)	4.4 (4-4.9)	5.1 (4.6-5.3)
**Viral load > 5 log**_**10**_	36%					

TDF, tenofovir; FTC, emtricitabine; ABC, abacavir; 3TC, lamivudine; EFV, efavirenz; ATV/r, atazanavir/ritonavir; LPV/r, lopinavir/ritonavir.

### Changes in immunovirological status during the first two years of effective cART

As requested, all the patients had plasma HIV viral loads below 400 copies/ml at M6 and below 50 copies/ml at M24. At M6, 72 patients (92%) had viral loads below 50 copies/ml. Between M6 and M24, twelve patients (15%) had isolated viral loads above 50 copies/ml (median 63 copies/ml; range 54 to 210). Among patients receiving TDF/FTC, 10 patients (16%) had isolated plasma viral loads above 50 copies/ml, compared to 2 patients (12%) receiving ABC/3TC (p = 0.64). Among patients receiving EFV, 3 patients (9%) had isolated viral loads above 50 copies/ml, compared to 4 patients (17%) receiving ATV/r and 5 patients (33%) receiving LPV/r (p = 0.08). During the 2-year study period, the CD4 cell count increased by 216/mm^3^ and the CD4/CD8 ratio by 0.42. At M24, the median CD4 cell count was 530/mm^3^ (IQR 393 to 683) and the median CD4/CD8 ratio was 0.76 (IQR 0.47 to 1.1). No patient developed AIDS defining event during the two years of treatment, while 11 patients experienced a non-AIDS defining event, consisting of pneumonia (n = 5), nephropathy (n = 4), osteoporosis (n = 1) and idiopathic gout (n = 1). None of the 28 smoking individuals quitted smoking while on treatment.

### Changes in markers of inflammation and immune activation during the first two years of effective cART

At baseline, the only difference in plasma biomarkers levels across the treatment groups was a higher IL-6 level in patients prescribed LPV/r (median 2.17 pg/ml) than in patients prescribed ATV/r or EFV (median 1.2 and 1.6 pg/mL, respectively; p = 0.040).

At M24, IL-6, IP-10 and MIG levels were significantly lower than at baseline (-40%, -59% and -74%, respectively), while sCD14 levels were unchanged (Table 2). The hs-CRP level fell by 23% but the change was not statistically significant.

**Table 2:** **Changes from M0 to M24 in plasma markers of inflammation and immune activation (log**_**e**_**-transformed values)**

		**Total n = 78**	**TDF/ FTC n = 61**	**ABC/ 3TC n = 17**	**EFV n = 36**	**ATV/r n = 27**	**LPV/r n = 15**
**IL-6**	Baseline (pg/ml)	1.6 (1.1-2.5)	1.6 (1.1-2.6)	1.2 (0.99-1.7)	1.6 (1.1-2.5)	1.2 (0.88-2.4)	2.17 (1.1-7.4)
	Mean fold change (95% CI)	0.60 (0.49, 0.74)	0.60 (0.48, 0.77)	0.60 (0.36, 0.99)	0.59 (0.42, 0.83)	0.82 (0.64, 1.05)	0.36 (0.20, 0.67)
	P value*	<0.001					
**hs-CRP**	Baseline (mg/L)	4 (2.2-15)	4.2 (2.3-15)	2.7 (1.8-9.4)	3.9 (2.2-17.8)	3 (2-5.9)	7.5 (2.9-20)
	Mean fold change (95% CI)	0.77 (0.57, 1.1)	0.81 (0.56, 1.20)	0.66 (0.33, 1.30)	0.76 (0.46, 1.26)	0.88 (0.54, 1.44)	0.63 (0.27, 1.47)
	P value	0.11					
**sCD14**	Baseline (10^6^ pg/ml)	2.6 (2.1-3.1)	2.6 (2.2-3.1)	2.5 (2-3.1)	2.5 (2.2-3)	2.6 (1.7-3.1)	3.1 (2-3.4)
	Mean fold change (95% CI)	1.00 (0.93, 1.1)	1.00 (0.93, 1.10)	0.96 (0.79, 1.15)	0.95 (0.85, 1.06)	1.10 (0.93, 1.26)	1.00 (0.85, 1.20)
	P value	0.82					
**IP-10**	Baseline (pg/ml)	664 (431-960)	630 (435-974)	721 (431-895)	692 (447-996)	549 (389-781)	798 (470-1326)
	Mean fold change (95% CI)	0.41 (0.35, 0.49)	0.44 (0.36, 0.52)	0.37 (0.27, 0.49)	0.34 (0.28, 0.42)	0.53 (0.40, 0.71)	0.43 (0.27, 0.67)
	P value	<0.001					
**MIG**	Baseline (pg/ml)	1532 (980-2804)	1534 (1004-2804)	1431 (938-2562)	1908 (1093-3799)	1182 (920-2201)	1611 (887-2444)
	Mean fold change (95% CI)	0.26 (0.19, 0.33)	0.25 (0.18, 0.35)	0.27 (0.17, 0.42)	0.16 (0.10, 0.24)	0.39 (0.30, 0.51)	0.39 (0.22, 0.70)
	P value	<0.001					

TDF, Tenofovir; FTC, Emtricitabine; ABC, Abacavir; 3TC, Lamivudine; EFV, Efavirenz; ATV/r, Atazanavir/ritonavir; LPV/r, Lopinavir/ritonavir; IL-6, Interleukin-6; hs-CRP, Highly sensitive C-reactive protein; sCD14, soluble CD14; 1P10, Interferon-γ-Inducible Protein 10; MIG, Monokine induced by interferon-γ; CI, Confidence Interval.

*One-sample Student’s *t* test of the change of each marker in the overall study sample.

### Impact of individual antiretroviral drugs on markers of inflammation and immune activation

Based on univariable linear regression models, between-group comparisons and the literature, the following variables were selected for multivariable analyses: age, smoking status, prior AIDS-defining events, baseline CD4 cell count, baseline viral load, hepatitis B or C virus coinfection, and viral blips above 50 copies/ml between M6 and M24. After adjustment for these variables and for the ART components (the NRTI backbone and the third agent), no significant difference in the decline in the IL-6 level was found between the TDF/FTC and ABC/3TC groups (mean fold change percentage difference ∆ 4%; p = 0.90) (Table 3). Compared to EFV, no significant difference in the change of IL-6 was associated with ATV/r or LPV/r use. In contrast, the choice of treatment regimen influenced the decline in IP-10 and MIG levels: the decline in both IP-10 (Δ = -57%, p = 0.011) and MIG (Δ = -136%; p = 0.007) was significantly smaller with ATV/r than with EFV, while no significant difference was found between LPV/r and EFV (IP-10 ∆ = -4%; p = 0.87; MIG ∆ = -48%; p = 0.44) or between ABC/3TC and TDF/FTC (IP-10 ∆ = 30%; p = 0.09; MIG Δ = 24%; p = 0.47).

**Table 3:** Regression analyses comparing the impact of antiretroviral therapy components on biomarker changes

		**Univariable**		Multivariable*	
**Marker**	**Antiretrovirals**	**Mean fold change percentage difference (95% CI)**	**P value**	**Mean fold change percentage difference (95% CI)**	**P value**
**IL-6**	TDF/FTC	Ref.			
	ABC/3TC	1 (-67 to 41)	0.97	4 (-80 to 49)	0.90
	EFV	Ref.			
	ATV/r	-39 (-120 to 12)	0.16	-20 (-101 to 28)	0.48
	LPV/r	38 (-8 to 63)	0.09	43 (-23 to 74)	0.15
**hs-CRP**	TDF/FTC	Ref.			
	ABC/3TC	18 (-77 to 62)	0.60	7 (-155 to 66)	0.88
	EFV	Ref.			
	ATV/r	-16 (-139 to 43)	0.68	-34 (-203 to 41)	0.47
	LPV/r	16 (-97 to 63)	0.67	41 (-29 to 82)	0.39
**sCD14**	TDF/FTC	Ref.			
	ABC/3TC	7 (-13 to 3)	0.48	14 (-8 to 31)	0.18
	EFV	Ref.			
	ATV/r	-14 (-36 to 4)	0.13	-19 (-43 to 1)	0.06
	LPV/r	-6 (-31 to 14)	0.57	-4 (-36 to 21)	0.77
**IP10**	TDF/FTC	Ref.			
	ABC/3TC	16 (-22 to 42)	0.36	30 (-6 to 54)	0.09
	EFV	Ref.			
	ATV/r	-55 (-118 to -12)	0.01	-57 (-120 to -11)	0.011
	LPV/r	-26 (-92 to 17)	0.27	-4 (-80 to 39)	0.87
**MIG**	TDF/FTC	Ref.			
	ABC/3TC	-8 (-103 to 42)	0.80	24 (-62 to 65)	0.47
	EFV	Ref.			
	ATV/r	-146 (-306 to -45)	0.001	-136 (-339 to -27)	0.007
	LPV/r	-151 (-395 to -31)	0.006	-48 (-297 to 45)	0.44

TDF, Tenofovir; FTC, Emtricitabine; ABC, Abacavir; 3TC, lamivudine; EFV, Efavirenz; ATV/r, Atazanavir/ritonavir; LPV/r, Lopinavir/ritonavir; IL6, Interleukin-6; hs-CRP, Highly-sensitive C-reactive protein; sCD14, soluble CD14; 1P10, Interferon-γ-inducible protein 10; MIG, Monokine induced by interferon-γ; CI, Confidence Interval.

*Adjusted for age, smoking, prior AIDS, baseline CD4 cell count, baseline HIV viral load, HCV or HBV coinfection, and viral blips.

No interaction was found between the NRTI backbone and the third agent for any of the marker, p-values of the interaction terms were 0.19, 0.26, 0.72, 0.34 and 0.91 for IL-6, hs-CRP, sCD14, IP-10 and MIG respectively. Similar results were obtained in sensitivity analyses that excluded patients with hepatitis virus coinfection.

## Discussion and conclusions

This observational study of changes in markers of inflammation and immune activation during the first two years of unchanged virologically effective first-line cART regimens (tenofovir- or abacavir-based NRTI backbone plus efavirenz, atazanavir/r or lopinavir/r) shows that the choice of the third antiretroviral agent influenced the degree of decline in markers of T-lymphocyte and macrophage activation. Plasma levels of IL-6, IP-10 and MIG fell by at least 40% in all the treatment groups, while the decline in hs-CRP levels failed to reach statistical significance and sCD14 levels were unchanged. The only observed difference between the treatment groups was that the T-lymphocyte and macrophage activation markers IP-10 and MIG fell less markedly in patients receiving ATV/r than in patients receiving EFV; no difference was found between LPV/r and EFV. Our patient population represents the naïve patients who initiated cART between January 2006 and December 2009 with a moderate immune deficit and a median viral load slightly below 100000 copies/ml and who experienced a rapid virological response in usual care in France.

Several aspects of study design are important in studying the influence of different antiretroviral drugs on biomarker variations in patients starting first-line therapy. First, it is important to control for viral replication, which is a major cause of persistent immunological activation and inflammation. As a result, the approach used in randomized trials, which includes all patients independently of their virological response, may not be appropriate. Furthermore, removal of patients with uncontrolled viral load from a randomized trial will result in a simple observational study. Despite the lack of randomization in our study, baseline characteristics were well balanced across the treatment groups, and factors that might have influenced the choice of cART regimen or the time course of the markers of interest were systematically included in the analyses. In addition, all the patients under care in the Infectious Diseases department of Pitié-Salpêtrière Hospital were screened for eligibility criteria, thus avoiding any major selection bias. However, one cannot exclude that unrecognized confounding regarding treatment selection could be present. In our study, the strict inclusion criteria regarding the viral replication resulted in a limited sample size. However, even with this sample size, significant differences were detected for some of the comparisons. In addition, most p-values of non-significant tests were above 0.15 indicating that for most comparisons, power was not an issue. It is possible that the freezing of plasma sample could have modified the marker levels and diminished our ability to detect differences. However, all the groups of antiretroviral regimens were studied similarly and this should not have biased the comparison between groups. Moreover, as in most published studies, studying thawed plasma allowed to study all marker dosages simultaneously in the same experiment, limiting the inter-individual variability. The high variability of inflammatory markers between individuals could have precluded seeing differences between treatment groups.

Elevated IL-6 and CRP levels before antiretroviral treatment initiation or after treatment interruption have been linked to a higher risk of AIDS-defining events and death [23,34]. In our study, IL-6 levels fell during the first two years of effective antiretroviral therapy, and the decline did not differ significantly between the NRTI backbones or across the different third agents. These results are consistent with those of the HEAT and ACTG A5224 trials [28,29]. With regard to hs-CRP, we found that its levels fell slightly although not significantly, with marked inter-individual differences and no differential effect of the studied antiretroviral drugs. In the ACTG A5224, HEAT and NICE trials, CRP levels either rose or fell after cART initiation, while a differential effect of antiretroviral drugs was found in ACTG A5224 trial but not in the HEAT or the NICE trials [28-30]. The high inter-individual variability, differences in study designs, such as discussed above, and in baseline characteristics may explain the discordant results.

Elevated plasma sCD14 levels are an independent predictor of death among HIV-infected patients [35]. SCD14, a marker of monocyte activation that correlates with HIV viremia, and is also considered to be a marker of microbial translocation across the intestinal mucosa, correlating positively with plasma lipopolysaccharide levels as being its soluble receptor [36,37]. Despite 2 years of effective antiretroviral therapy, sCD14 levels did not change significantly, in keeping with previous reports, suggesting that regimens used in our study may not restore the intestinal barrier function, resulting in persistent microbial translocation and immune activation [36,38]. Previous study has reported that among treated HIV-infected patients, persistent HIV-DNA in the gut correlates with levels of microbial translocation and immune activation [39]. Interestingly, Taiwo B et al. have recently reported that an NRTI-sparing regimen consisting of boosted darunavir plus raltegravir led to a decline in sCD14, IL-6 and IP-10 levels [40], suggesting that integrase inhibitors may be more effective in this respect than other antiretroviral drugs.

Persistent immune activation despite virologically effective therapy has been linked to immunological failure [41,42]. While IL-6, hs-CRP, sCD14 and IP-10 have been studied in other studies, MIG has not been evaluated in the context of naïve patients initiating cART. IP-10 (CXCL-10) and MIG (CXCL-9), two chemokines induced by interferon gamma, specifically target lymphocytes, particularly activated T cells, as well as macrophages, and are critical mediators of T cell migration during T-cell-dependent immune responses [43]. High levels of these chemokines reflect immunological activation in HIV-infected patients [44]. A recent study has shown that elevated plasma IP-10 levels in primary HIV-1 infection are strongly predictive of rapid HIV disease progression [45]. In our study, IP-10 and MIG levels showed the largest decline among the studied biomarkers during the first two years of effective cART, running parallel to the fall in plasma viremia. IP-10 and MIG were also the only markers for which a differential effect of the studied antiretroviral drugs was observed. The fall in both markers was larger with EFV than with ATV/r. To investigate whether these differences could be explained by the change in CD4 cell count, analyses were made adjusting for the change of CD4 cell count and differences were still significant and could not be explained by the change in CD4 cell count. We hypothesize that other ART-mediated mechanisms such as the more rapid decay in HIV-RNA in the first 14 days of treatment initiation associated with EFV than with ATV/r observed in a randomized study [46] could explain, at least partly, the observed difference between EFV and ATV/r. However, it is unclear whether this has any clinical implications. To our knowledge, the differential impact of antiretroviral drugs on IP-10 and MIG has not previously been studied in naïve HIV-infected patients.

The differential effect of the studied antiretroviral drugs on changes of two immune activation markers such as IP-10 and MIG levels suggests that these markers could be worthwhile when evaluating new antiretroviral drugs. The presence of sCD14 despite two years of viral suppression reflects the persistence of immune activation, and may contribute to the maintenance of residual viremia and HIV DNA reservoirs [47]. With the growing interest for finding a cure for HIV infection and with the preoccupation of long-term management of HIV-infected individuals, it is key to assess the capacity of new drugs and new combinations of drugs to drive immune activation and inflammation down.

## Competing interests

No members of the study team have any financial or personal relationships with people or organizations that could inappropriately influence this work. Dominique Costagliola and Christine Katlama have received at some stage in the past travel grants, consultancy fees, honoraria and study grants from various pharmaceutical companies including Bristol-Myers-Squibb, Gilead Sciences, Janssen-Cilag, Merck-Sharp & Dohme-Chibret and ViiV Healthcare.

## Authors’ contributions

Conception and design: SH, AG, MG, GC, A-GM, DC, CK. Plasma samples collection: SH, SF, A-GM. Biomarkers measurements: SH, AG, GC, BA. Collection and assembly of data: SH, AG, MG, SF, FC. Statistical analyses: SH, MG, DC. Critical revision of the article for scientific accuracy: all. Final approval of the article: all.

## Pre-publication history

The pre-publication history for this paper can be accessed here:

http://www.biomedcentral.com/1471-2334/14/122/prepub
